# Short-Term Comparative Effectiveness of Intra-articular Corticosteroid Injection Versus Hydrostatic Distention in Idiopathic Frozen Shoulder: A Prospective Interventional Study

**DOI:** 10.7759/cureus.86639

**Published:** 2025-06-24

**Authors:** Muhammad Anas Ghazi, Slah Ud Din, Zunair Aqeel, Zia Ullah, Tauseef Raza, Kashif Anwar, Mohammed Qasim Rauf, Franklin E Ehizojie, Aimal K Sattar, Hafiz Ali Raza

**Affiliations:** 1 Trauma and Orthopaedics, North Manchester General Hospital, Manchester, GBR; 2 Orthopaedics, Health and Population, Tehsil Headquarter Hospital Kot Chutta, Dera Ghazi Khan, PAK; 3 Surgery, Ghurki Trust Teaching Hospital, Lahore, PAK; 4 Orthopaedics, Khalifa Gul Nawaz Teaching Hospital, Bannu Medical College, Bannu, PAK; 5 Orthopaedics, KMU (Khyber Medical University) Institute of Medical Sciences, Kohat, PAK; 6 Orthopaedics, Jinnah Postgraduate Medical Centre, Karachi, PAK; 7 Orthopaedic Surgery, The Hillingdon Hospitals NHS Foundation Trust, London, GBR; 8 Health Sciences, Purdue University, West Lafayette, USA; 9 Orthopaedic Surgery, Lady Reading Hospital, Peshawar, PAK; 10 Agriculture Extension, Muhammad Nawaz Shareef University of Agriculture, Multan, PAK

**Keywords:** corticosteroid injection, hydrostatic distention, idiopathic frozen shoulder, pain relief, shoulder function, spadi, vas

## Abstract

Background

Idiopathic frozen shoulder (adhesive capsulitis) is a common and debilitating condition, characterized by progressive restriction of shoulder movement. Non-randomized interventional treatments, such as intra-articular corticosteroid injections and hydrostatic (hydrodilatation) distention, are commonly employed when conservative therapy fails.

Objective

The main objective of this study is to compare the short-term effectiveness of intra-articular corticosteroid injection versus hydrostatic distention, in terms of pain relief and functional improvement in patients with idiopathic frozen shoulder.

Methods

This prospective, single-center, comparative interventional study was conducted at Lady Reading Hospital, Peshawar, Pakistan. A total of 108 patients, aged 35-70 years, with frozen-phase idiopathic frozen shoulder (>3 months’ duration), were assigned to two treatment groups using non-random, consecutive allocation: Group A (n = 54) received an intra-articular corticosteroid injection, and Group B (n = 54) underwent hydrostatic shoulder distention. Patients were assessed at baseline, 4 weeks, and 12 weeks using the Visual Analog Scale (VAS) for pain and the Shoulder Pain and Disability Index (SPADI) for function. Statistical analysis was performed using IBM SPSS Statistics for Windows, Version 25 (Released 2017; IBM Corp., Armonk, NY, USA).

Results

Both groups showed significant improvements in pain and function over time (p < 0.001). However, Group A demonstrated superior outcomes at 12 weeks in VAS (2.1 ± 0.9 vs. 2.6 ± 1.0; p = 0.027) and SPADI (28.6 ± 6.3 vs. 32.9 ± 6.5; p = 0.006). “Very satisfied” patients were more frequent in Group A (28 patients; 64.81%) than in Group B (35 patients; 51.85%).

Conclusion

Intra-articular corticosteroid injection appears to be more effective than hydrostatic shoulder distention in providing short-term pain relief, functional improvement, and higher patient satisfaction in idiopathic frozen shoulder. Further studies, with randomized designs and long-term follow-up, are warranted.

## Introduction

Frozen shoulder, also known as idiopathic frozen shoulder, or historically referred to as adhesive capsulitis, is a debilitating condition marked by progressive shoulder pain and significant restriction of both active and passive range of motion [1,2]. Recent literature suggests that the term "frozen shoulder" is preferable, as alternatives such as "adhesive capsulitis," "capsular contracture," or "retractile capsulitis" may not fully capture the clinical complexity or variability of the condition [3,4]. Although frozen shoulder is often self-limiting, its course may extend over months or even years, substantially impairing daily activities and quality of life [5].

While its exact etiology remains uncertain, it is widely believed to involve chronic low-grade inflammation of the synovial tissue, followed by fibrosis and contracture of the joint capsule, leading to pain and stiffness [6]. Ryan et al. detailed these pathophysiological mechanisms in a comprehensive systematic review [6]. The natural history of the disease is classically divided into three overlapping stages: freezing, frozen, and thawing [3]. In the absence of early intervention, recovery may be prolonged and incomplete, prompting clinicians to seek effective therapeutic strategies that can hasten symptom resolution and restore function [4].

Idiopathic frozen shoulder can be managed in a variety of ways, from conservative measures like physical therapy and analgesics to more invasive techniques such as arthroscopic release, hydraulic distention, intra-articular corticosteroid injections, and manipulation under anesthesia [7,8]. Among these, hydrostatic shoulder distention and corticosteroid injections have drawn special interest due to their minimally invasive nature and ability to alleviate symptoms in the condition’s early and middle phases [9].

Injections of intra-articular corticosteroids are often used to relieve pain, restore range of motion, and lessen joint inflammation. It is believed that these injections prevent capsular fibrosis and reduce inflammatory cytokines [10]. In contrast, hydrostatic distention involves injecting a fluid - often saline or a steroid-and-saline mixture - under controlled pressure into the glenohumeral joint to expand the capsule and break down adhesions, thereby improving mobility [11]. The relative effectiveness of the two approaches is still in question, despite evidence of varying degrees of success, especially in terms of functional outcomes, pain relief, and duration of therapeutic benefit [12].

Finding the best treatment option is crucial for providing the best possible care for patients with frozen shoulders, since it limits their movement and freedom. Despite a large amount of research, reaching a clear agreement remains difficult, and there is little comparative data available for specific groups.

Research objective

The main objective is to compare the clinical effectiveness of intra-articular corticosteroid injection versus hydrostatic shoulder distention in the treatment of idiopathic frozen shoulder in terms of pain relief and improvement in shoulder function.

## Materials and methods

Study design and setting

This prospective, single-center, comparative interventional study was conducted in the Department of Orthopaedic Surgery at Lady Reading Hospital, Peshawar, Pakistan, over a one-year period, from January 2024 to December 2024. The study adhered to the Transparent Reporting of Evaluations with Nonrandomized Designs (TREND) statement, which is suitable for non-randomized interventional studies [13,14].

Inclusion and exclusion criteria

Patients between the ages of 35 and 70, who had been diagnosed with idiopathic frozen shoulder (adhesive capsulitis) in the frozen phase, and who had experienced restricted shoulder movement for more than three months, were included. To rule out additional shoulder diseases, radiographic imaging was used to confirm the diagnosis, which was based on clinical examination.

Patients with secondary frozen shoulder brought on by trauma, surgery, or systemic illnesses such as diabetes mellitus, thyroid issues, or autoimmune diseases were excluded. Additionally, patients with significant osteoarthritis, rotator cuff injuries, previous shoulder injections, and contraindications to corticosteroids or distention operations were also excluded.

Sample size

A total of 108 patients meeting the eligibility criteria were enrolled using a convenience sampling technique. Patients were assigned to two groups using non-random, consecutive allocation based on clinical scheduling and appointment availability: Group A (n = 54) received intra-articular corticosteroid injections, and Group B (n = 54) underwent hydrostatic shoulder distention. Although a formal power calculation was not performed due to feasibility constraints and limited comparative data, steps were taken to reduce the risk of Type II error. These included ensuring equal group sizes, using validated outcome measures (Visual Analog Scale, or VAS, and Shoulder Pain and Disability Index, or SPADI), and employing sensitive statistical methods [15-17]. All patients were informed of both treatment options, and written informed consent was obtained prior to enrollment.

Data collection

At enrollment, baseline demographic information, clinical history, and shoulder function were documented. Body Mass Index (BMI) was calculated using the standard formula (weight in kilograms divided by height in meters squared). The VAS was used to measure the severity of pain, and the SPADI was used to measure shoulder function. At baseline, 4 weeks, and 12 weeks after the intervention, both outcomes were assessed. Using the posterior technique on the glenohumeral joint, Group A received an aseptic intra-articular injection of 40 mg (1 mL) of triamcinolone acetonide and 1 mL of 1% lidocaine hydrochloride. Triamcinolone was selected for its long intra-articular half-life and favorable safety and efficacy profile, as supported by existing literature [18]. Up to 40 mL of normal saline, combined with 40 mg of triamcinolone acetonide, was administered to Group B by hydrostatic distention under ultrasound or fluoroscopic supervision until resistance was removed - defined as a subjective pressure drop and observable improvement in passive motion, suggesting capsular stretching and possible adhesion rupture.

Statistical analysis

IBM SPSS Statistics for Windows, Version 25 (Released 2017; IBM Corp., Armonk, NY, USA), was used to analyze the data. Baseline characteristics were summarized using descriptive statistics (mean, standard deviation, frequency, and percentage). Independent sample t-tests were used to compare continuous variables (VAS and SPADI scores) across groups. Paired t-tests were used to examine changes over time within the groups. Statistical significance was defined as a p-value of less than 0.05.

Ethical approval

**Figure 1:**
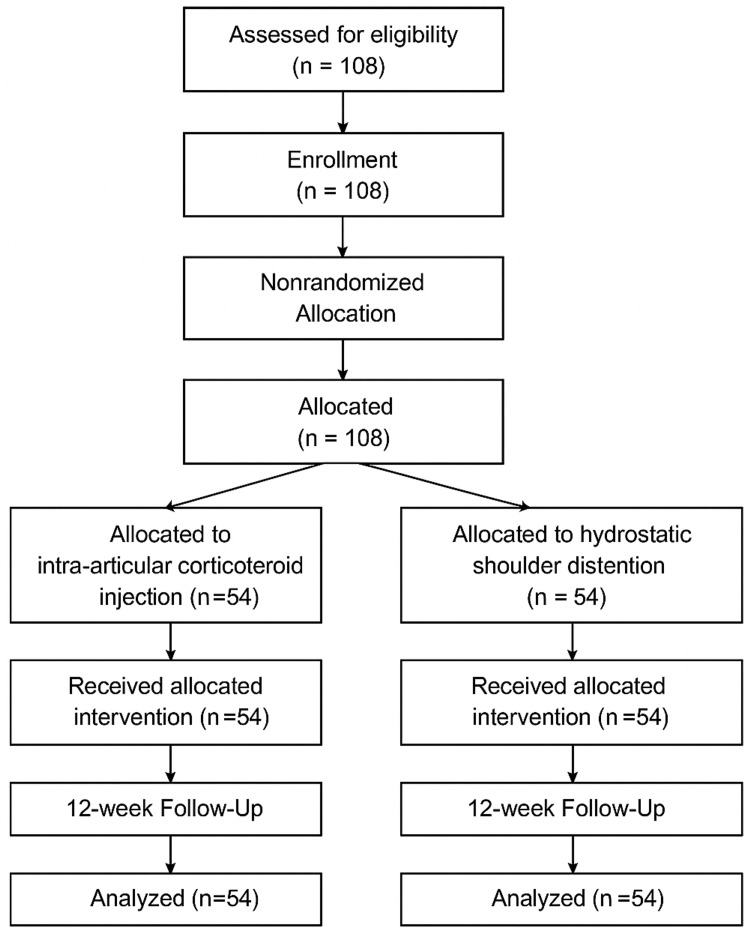
Participant Flow Diagram

The study was approved by the Institutional Review Board (IRB) of Lady Reading Hospital, Peshawar. A TREND-style participant flow diagram (Figure 1) has been included to outline patient progression through the study, including screening, enrollment, allocation, follow-up, and final analysis. No patients were lost to follow-up during the 12-week period.

## Results

Table 1 presents the baseline demographic characteristics of the 108 patients enrolled in the study, with an equal distribution between Group A (steroid injection) and Group B (hydrostatic distention), each comprising 54 individuals. The mean age was 54.2 ± 8.5 years in Group A and 53.6 ± 9.1 years in Group B. Males accounted for 28 (51.85%) patients in Group A and 25 (46.30%) in Group B. The mean BMI was 27.3 ± 2.8 kg/m² in Group A and 27.7 ± 3.1 kg/m² in Group B, with no statistically significant difference between the two groups (p = 0.314). The right shoulder was more frequently affected in both groups, observed in 30 (55.56%) patients in Group A and 32 (59.26%) in Group B. This unusual pattern might be due to the specific age and job types of the patients at Lady Reading Hospital in Peshawar, where many male patients who do physical work come for treatment. Symptom duration ranged from three to six months for most patients - 33 (61.11%) in Group A and 35 (64.81%) in Group B - while 21 (38.89%) and 19 (35.19%) patients in Groups A and B, respectively, reported symptoms lasting longer than six months.

**Table 1:** Demographic Characteristics of Patients (N = 108) *p = 0.314; p-values less than 0.05 were considered statistically significant.

Category	Characteristic	Group A: Steroid Injection (n, %)	Group B: Hydrostatic Distention (n, %)
Age (years)	Mean ± SD	54.2 ± 8.5	53.6 ± 9.1
Gender	Male	28 (51.85)	25 (46.30)
	Female	26 (48.15)	29 (53.70)
BMI (kg/m²)*	Mean ± SD	27.3 ± 2.8	27.7 ± 3.1
Affected Side	Right Shoulder	30 (55.56)	32 (59.26)
	Left Shoulder	24 (44.44)	22 (40.74)
Symptom Duration	3-6 months	33 (61.11)	35 (64.81)
	>6 months	21 (38.89)	19 (35.19)

Table 2 contrasts baseline, 4-week, and 12-week pain (VAS) and function (SPADI) ratings. Over time, both groups made progress, although Group A's improvement was statistically larger. Group A's VAS was 4.3 ± 1.1 at four weeks, whereas Group B's was 4.8 ± 1.2 (p = 0.038); after 12 weeks, it had decreased to 2.1 ± 0.9 vs. 2.6 ± 1.0 (p = 0.027). Likewise, after four weeks, Group A's SPADI was 49.2 ± 7.5 compared to Group B's 53.1 ± 8.1 (p = 0.014), and at 12 weeks, it was 28.6 ± 6.3 compared to 32.9 ± 6.5 (p = 0.006).

**Table 2:** Comparison of Outcomes Within Groups Over Time *p-values less than 0.05 were considered statistically significant. VAS, Visual Analog Scale; SPADI, Shoulder Pain and Disability Index

Outcome Measure	Time Point	Group A: Mean ± SD	Group B: Mean ± SD	p-value (Between Groups)
VAS Score	Baseline	7.9 ± 0.8	8.0 ± 0.7	0.472
	4 weeks	4.3 ± 1.1	4.8 ± 1.2	0.038*
	12 weeks	2.1 ± 0.9	2.6 ± 1.0	0.027*
SPADI Score	Baseline	75.3 ± 6.2	74.6 ± 6.8	0.591
	4 weeks	49.2 ± 7.5	53.1 ± 8.1	0.014*
	12 weeks	28.6 ± 6.3	32.9 ± 6.5	0.006*

**Figure 2:**
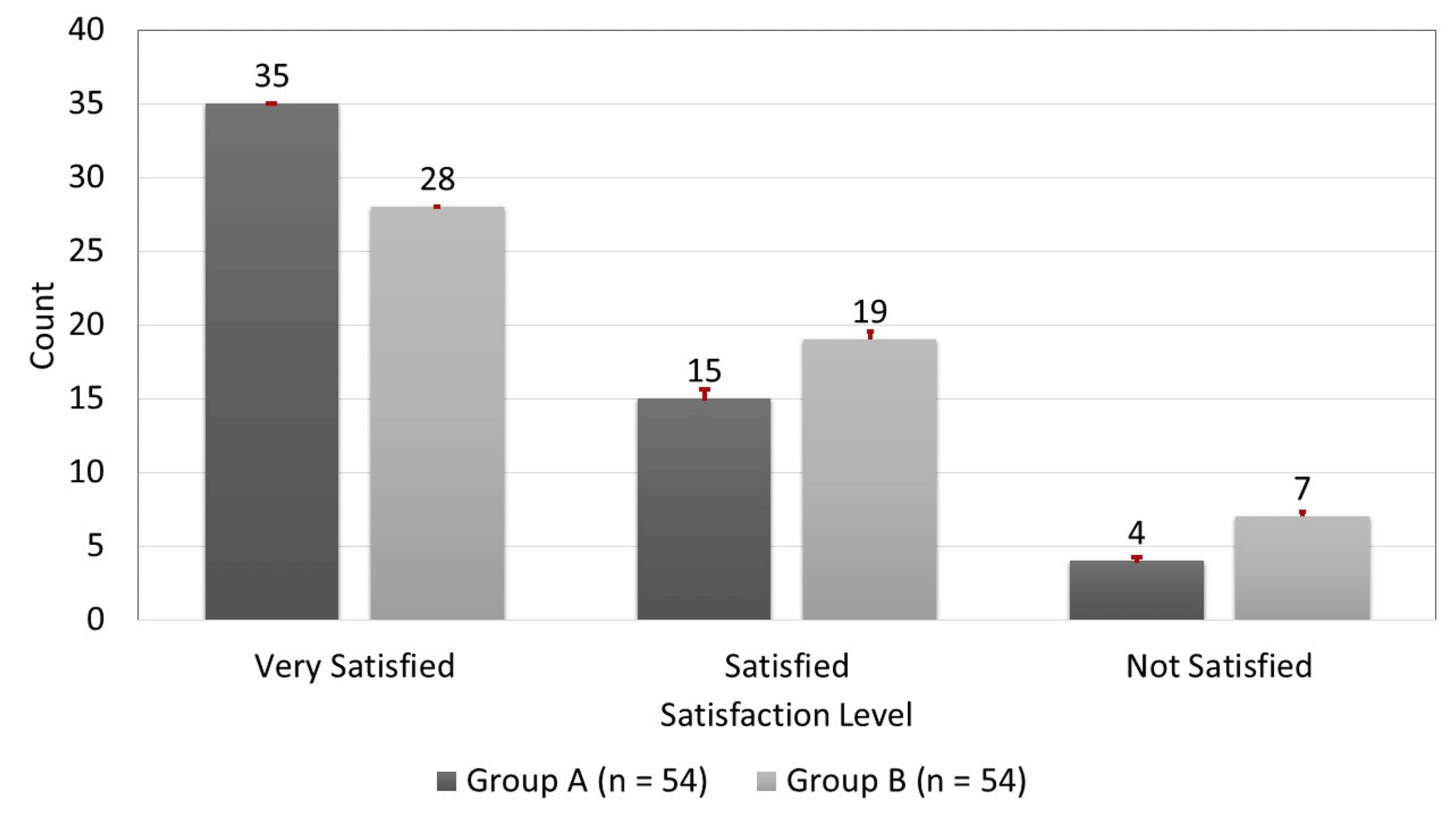
Patient-Reported Satisfaction at 12 Weeks Red dots show the percentage.

Figure 2 summarizes patient-reported satisfaction at 12 weeks. A higher proportion of patients in Group A were "very satisfied": 35 (64.81%) compared to 28 (51.85%) in Group B. Satisfaction was reported by 15 (27.78%) patients in Group A and 19 (35.19%) in Group B. Meanwhile, four (7.41%) patients in Group A and seven (12.96%) in Group B expressed dissatisfaction with the treatment outcomes.

Statistical comparisons between groups are shown in Table 3. There were no discernible differences at baseline (VAS, p = 0.472; SPADI, p = 0.591). Group A consistently showed significantly greater improvement between 4 and 12 weeks. For instance, after 12 weeks, Group A's SPADI was 28.6 ± 6.3, whereas Group B's was 32.9 ± 6.5 (mean difference: -4.3, p = 0.006).

**Table 3:** Independent Sample t-test Comparing VAS and SPADI Scores Between Groups *p-values less than 0.05 were considered statistically significant. VAS, Visual Analog Scale; SPADI, Shoulder Pain and Disability Index

Time Point	Outcome Measure	Mean ± SD (Group A)	Mean ± SD (Group B)	Mean Difference	t-value	p-value
Baseline	VAS	7.9 ± 0.8	8.0 ± 0.7	-0.1	-0.72	0.472
	SPADI	75.3 ± 6.2	74.6 ± 6.8	0.7	0.54	0.591
4 Weeks	VAS	4.3 ± 1.1	4.8 ± 1.2	-0.5	-2.11	0.038*
	SPADI	49.2 ± 7.5	53.1 ± 8.1	-3.9	-2.51	0.014*
12 Weeks	VAS	2.1 ± 0.9	2.6 ± 1.0	-0.5	-2.24	0.027*
	SPADI	28.6 ± 6.3	32.9 ± 6.5	-4.3	-2.83	0.006*

Table 4 shows improvements within the group. Over the 12-week period, Group A's VAS dropped from 7.9 to 2.1, and its SPADI from 75.3 to 28.6 (both p < 0.001). Group A showed somewhat larger overall decreases, while Group B also made considerable progress (VAS from 8.0 to 2.6 and SPADI from 74.6 to 32.9).

**Table 4:** Paired t-test Analysis of VAS and SPADI Scores Within Each Group Over Time *p-values less than 0.05 were considered statistically significant. VAS, Visual Analog Scale; SPADI, Shoulder Pain and Disability Index

Group	Outcome Measure	Time Points	Mean ± SD	Mean Difference	t-value	p-value
Group A (Steroid Injection)	VAS	Baseline vs. 4 weeks	7.9 ± 0.8 → 4.3 ± 1.1	-3.6	18.74	<0.001*
	VAS	4 weeks vs. 12 weeks	4.3 ± 1.1 → 2.1 ± 0.9	-2.2	15.26	<0.001*
	SPADI	Baseline vs. 4 weeks	75.3 ± 6.2 → 49.2 ± 7.5	-26.1	21.45	<0.001*
	SPADI	4 weeks vs. 12 weeks	49.2 ± 7.5 → 28.6 ± 6.3	-20.6	19.18	<0.001*
Group B (Hydrostatic Distention)	VAS	Baseline vs. 4 weeks	8.0 ± 0.7 → 4.8 ± 1.2	-3.2	17.31	<0.001*
	VAS	4 weeks vs. 12 weeks	4.8 ± 1.2 → 2.6 ± 1.0	-2.2	13.49	<0.001*
	SPADI	Baseline vs. 4 weeks	74.6 ± 6.8 → 53.1 ± 8.1	-21.5	18.92	<0.001*
	SPADI	4 weeks vs. 12 weeks	53.1 ± 8.1 → 32.9 ± 6.5	-20.2	17.85	<0.001*

## Discussion

The present study compared the short-term clinical efficacy of hydrostatic shoulder distention and intra-articular corticosteroid injections in treating idiopathic frozen shoulder, with an emphasis on improving shoulder function and reducing pain. Both interventions produced statistically significant improvements over time; however, the intra-articular corticosteroid injection showed superior outcomes, particularly in pain relief and functional recovery over the 12-week follow-up period.

In terms of pain reduction, Group B (hydrostatic distention) exhibited a decrease in mean VAS score from 8.0 ± 0.7 to 2.6 ± 1.0 (p < 0.001), while Group A (steroid injection) improved from 7.9 ± 0.8 at baseline to 2.1 ± 0.9 at 12 weeks (p < 0.001). Group A showed statistically significant advantages over Group B at both 4 and 12 weeks (p = 0.038 and p = 0.027, respectively). These findings align with recent systematic reviews and meta-analyses, which report that corticosteroid injections offer superior short-term pain control in frozen shoulder, especially during the frozen phase [19-21].

Bryant et al. [22] reported that, while hydrostatic distention can be beneficial, its analgesic effects were typically less robust than those of corticosteroid injections. A 2022 meta-analysis by Jain et al. further emphasized that intra-articular steroids provide faster and more pronounced pain relief compared to distention or physical therapy alone [23]. Our findings reinforce the clinical preference for corticosteroids when rapid symptom control is a priority.

Regarding shoulder function, both groups showed marked improvement, but Group A demonstrated a greater reduction in SPADI scores - from 75.3 ± 6.2 to 28.6 ± 6.3 (p < 0.001) - compared to Group B, which improved from 74.6 ± 6.8 to 32.9 ± 6.5 (p < 0.001). The between-group difference at 12 weeks was statistically significant (mean difference: -4.3, p = 0.006). These results are consistent with recent high-quality trials and research studies showing that corticosteroid injections lead to greater functional recovery in idiopathic frozen shoulder compared to other modalities [24-26]. Although hydrostatic distention contributed to functional gains, it was less effective overall. A recent study confirmed similar findings, noting that while hydrodilatation improves range of motion, corticosteroids result in superior short-term function and pain outcomes when used early in the frozen phase [27].

Strengths and limitations of the study

Patient-reported satisfaction was notably higher in the corticosteroid group, with 64.81% of patients reporting high satisfaction compared to 51.85% in the hydrostatic distention group. This likely reflects the faster and more pronounced symptom relief achieved with steroid injections. However, patient expectations or placebo effects - particularly in an unblinded study design - may have influenced satisfaction, given its inherently subjective nature. This limitation is important to consider and has been acknowledged in the limitations section. Although both treatments demonstrated clinical effectiveness over the 12-week follow-up, no conclusions regarding long-term efficacy were drawn, as the limited duration may not fully capture relapses or late-phase recovery. Further research with extended follow-up is warranted to evaluate the durability and sustained functional outcomes of both interventions.

This study's strengths include its prospective design, standardized interventions, and use of validated outcome measures (VAS and SPADI), which enhance internal validity. Clear inclusion and exclusion criteria reduced confounding, while balanced group sizes (n = 54 each) and appropriate statistical tests (paired and independent sample t-tests) ensured robust comparisons. Though no formal power analysis was conducted, a moderate sample size based on one year of patient volume was used, and steps were taken to reduce Type II error by employing sensitive instruments and ensuring group comparability. Blinded outcome assessments and consistent procedural protocols further minimized bias. Nonetheless, limitations remain: the single-center design and convenience sampling may introduce selection bias and limit generalizability. The 12-week timeframe does not capture long-term outcomes. While patient-reported outcomes offer clinical relevance, they remain vulnerable to subjective interpretation and bias, which may influence satisfaction ratings and perceived improvement.

## Conclusions

In summary, this research shows that, for individuals with idiopathic frozen shoulder, intra-articular corticosteroid injections are superior to hydrostatic shoulder distention in terms of reducing pain and enhancing shoulder function. The data show that the corticosteroid injection group experienced better levels of pain relief, functional improvement, and patient satisfaction. In the short term, corticosteroid injections appear to provide more rapid and longer-lasting effects, even though both therapies are effective. Further evaluation of the long-term safety and effectiveness of these therapies in a broader and more diverse population will require additional studies with multicenter data and longer follow-up.
